# Identification of Small-Molecule Inhibitors of *Brucella* Diaminopimelate Decarboxylase by Using a High-Throughput Screening Assay

**DOI:** 10.3389/fmicb.2019.02936

**Published:** 2020-01-21

**Authors:** Pengfei Bie, Xiaowen Yang, Cunrui Zhang, Qingmin Wu

**Affiliations:** Department of Preventive Veterinary Medicine, College of Veterinary Medicine, China Agricultural University, Beijing, China

**Keywords:** diaminopimelate decarboxylase, saccharopine dehydrogenase, lysine biosynthesis, high-throughput screening, enzyme inhibitors

## Abstract

Brucellosis, caused by intracellular gram-negative pathogens of the genus *Brucella*, continues to be one of the most pandemic zoonotic diseases in most countries. At present, the therapeutic treatment of brucellosis relies on a combination of multiple antibiotics that involves a long course of treatment, easy relapse, and high side effects from the use of certain antibiotics (such as streptomycin). Thus, the need to identify novel drugs or targets to control this disease is urgent. Diaminopimelate decarboxylase (DAPDC), a key enzyme involved in the bacterial diaminopimelate (DAP) biosynthetic pathway, was suggested to be a promising anti-*Brucella* target in our previous study. In this work, the biological activity of *Brucella melitensis* DAPDC was characterized, and a library of 1,591 compounds was screened for inhibitors of DAPDC. The results of a high-throughput screening (HTS) assay showed that 24 compounds inhibited DAPDC activity. In a further *in vitro* bacterial inhibition experiment, five compounds exhibited anti-*Brucella* activity (SID3, SID4, SID14, SID15, and SID20). These results suggested that the identified compounds can be used as potent molecules against brucellosis and that the application ranges of these approved drugs can be expanded in the future.

## Introduction

The *Brucella* spp. are gram-negative, facultative intracellular pathogens that cause brucellosis, which afflicts over 500,000 people annually and severely damages animal production (Godfroid et al., 2005; Seleem et al., 2010). At present, the genus *Brucella* contains 10 species, among which four species (*Brucella melitensis, Brucella suis*, *Brucella abortus*, and *Brucella canis*) are thought to be pathogenic to humans (Rajashekara et al., 2004; Godfroid et al., 2011). Moreover, *B. melitensis, B. suis*, and *B. abortus* are also the main causes of human brucellosis due to their highly contagious characteristics and are listed as potential bioweapons by the Centers for Disease Control and Prevention (CDC) (Chain et al., 2005). These organisms cause abortion, infertility, and low milk production in their natural hosts (bovines, goats, and sheep), whereas the common manifestations of human infection include undulant fever, arthralgia, asthenia, depression, and lymphadenopathy; therefore, these organisms pose a substantial threat to livestock production, food safety, and human health (Hasanjani and Ebrahimpour, 2015).

*Brucella* can survive and replicate in different types of host cells and establish intracellular replicative niches that protect them from the immune responses of the host after infection. In view of these characteristics, the clinical treatment of brucellosis has become difficult, and drug combination regimens of two or more antibiotics (e.g., a combination of doxycycline and streptomycin) are recommended to treat *Brucella* infections that still have a potential risk of treatment failure and relapse (Alp et al., 2006; Yousefi-Nooraie et al., 2012). Furthermore, no effective human vaccine to prevent human infection is available, so there is an urgent need to design and develop new drugs to treat brucellosis.

The enzymes that play critical roles in essential metabolic pathways have always attracted the attention of scientists in the field of drug discovery. The widely used first-line brucellosis drug rifampicin exhibits bacteriostatic activity via selective binding to the β subunit of DNA-dependent RNA polymerase and further inhibiting the synthesis of bacterial DNA and protein (Pang et al., 2013). Serer et al. (2019) reported a series of anti-*Brucella* compounds that share a similar chemical scaffold (2-phenylamidazo [2, 1-b] [1, 3] benzothiazole) via an HTS assay of 44,000 compounds targeting riboflavin synthase, which is essential to the riboflavin pathway. DAPDC encoded by the *lysA* gene (BMNI I1887) is a PLP-dependent enzyme that catalyzes the irreversible decarboxylation of meso-DAP to yield L-lysine, the final step of the lysine biosynthetic pathway in bacteria and plants. L-lysine, the product of the enzymatic reaction, is itself a fundamental component of the biosynthesis of housekeeping proteins and virulent factors in bacteria (Hutton et al., 2003; Peverelli and Perugini, 2015). In microbiology, the precursor of L-lysine, DAP, is also an important component in the peptidoglycan layer of bacterial cell walls, providing resistance to osmotic pressure and maintaining cell integrity (Meroueh et al., 2006). More importantly, L-lysine is an essential amino acid that cannot be biosynthesized by mammals, including humans, and must be obtained from the diet, making L-lysine biosynthetic enzymes promising antibacterial drug targets.

In our previous study, a *B. melitensis* transposon mutant library containing 32,640 transposon mutants was established and sequenced to identify transposon insertion sites. A total of 948 genes without transposon insertions in *B. melitensis* genomes, one of which was the *lysA* gene, were analyzed and listed as potential essential genes (De et al., 2017). To further validate the essentiality of the *lysA* gene in *Brucella* survival, we tried to replace the target gene in the *B. melitensis* genome with a kanamycin resistance gene via homologous recombination but failed to obtain the deficient phenotype after several attempts.

In this study, we developed an HTS assay to screen inhibitors of *B. melitensis* DAPDC. Five of the primary hits were confirmed in a minimum inhibitory concentration (MIC) assay to have bactericidal activity against *B. melitensis*, suggesting that the *lysA* gene might be essential for *Brucella* survival. The identified compounds might serve as a starting point for the development of potent anti-*Brucella* drugs.

## Materials and Methods

### Reagents, Strains, Plasmids, and Growth Conditions

Unless mentioned, the reagents used in this study were purchased from Sigma-Aldrich (Merck, Germany). A small-molecule drug library consisting of 1,591 drugs approved by the US Food and Drug Administration (FDA) was purchased from Selleck (Selleck Chemicals, United States).

The pET21b and pET30a plasmids were used as expression vectors. *Escherichia coli* strains DH5α and BL21 (DE3) were selected as hosts for cloning and protein expression, respectively. The *B. melitensis* NI strains that were first isolated from an aborted bovine fetus by our laboratory were used as hosts for construction of a marked *lysA* gene deletion mutant and provided the genomic DNA used to amplify the *lysA* gene for construction of pET21b-*lysA* vector, and *B. melitensis* 16M strains were used for MIC determination. Various *E. coli* and *Brucella* strains were grown in Luria-Bertani (LB) medium and Mueller-Hinton broth (Oxoid, United Kingdom), respectively. Ampicillin and kanamycin were used at a final concentration of 100 μg/ml.

### Construction of a Marked Deletion Mutant of the DAPDC Gene

A gene deletion construct was constructed as described with some modifications (Dong et al., 2013). The primers used for gene deletion are shown in Supplementary Table S1. Briefly, the sequence upstream (500 bp) of the *lysA* gene was amplified from the *B. melitensis* NI genome via polymerase chain reaction (PCR) using the primer pair Upstream-F and Upstream-R. The sequence downstream (500 bp) of the *lysA* gene was amplified using the primer pair Downstream-F and Downstream-R. Then, overlapping PCR was performed to ligate the two homologous arms of the *lysA* gene, and the overlap product was cloned into the suicide plasmid pBluescript II KS (+) via *Sac*I and *Bam*HI sites. Subsequently, a kanamycin resistance (Kan^r^) DNA fragment amplified from the pKD4 plasmid was subcloned into the plasmid with a unique *Xba*I site. The resulting construct was then introduced into *B. melitensis* NI cells via electroporation. The potential marked deletion mutant was selected in the presence of 100 μg/ml kanamycin and verified by PCR amplification using the primer pair Upstream-F and Downstream-R.

### Multiple Sequence Alignment and Phylogenetic Analysis of DAPDC Homologs and Eukaryotic Ornithine Decarboxylase (ODC) Enzymes

The amino acid sequences of DAPDC homologs and eukaryotic ODC enzymes from different species were obtained from the National Center for Biotechnology Information protein database. Then, the protein sequence of *B. melitensis* DAPDC was compared with that of the other obtained DAPDC homologs and eukaryotic ODC enzymes using the BLASTP program on the website http://blast.ncbi.nlm.nih.gov/Blast.cgi. ClustalW software was used for multiple sequence alignment analysis, and the results were edited on the website http://espript.ibcp.fr/ESPript/cgi-bin/ESPript.cgi. Phylogenetic analysis was performed using MEGA 5.0 software with neighbor-joining methods.

### Expression and Purification of *B. melitensis* DAPDC

The *lysA* gene was amplified from the genomic DNA of *B. melitensis* NI by PCR. Sequences of the primers used to construct the pET21b-*lysA* vector are shown in Supplementary Table S1. Then, PCR products were cleaved with *Bam*HI and *Xho*I restriction enzymes and inserted into the pET21b with the same restriction sites, producing a recombinant expression vector containing the DAPDC protein and an N-terminal hexahistidine tag. After confirming sequence insertion by DNA sequencing, the pET21b-*lysA* vector was transformed into *E. coli* BL21 competent cells.

To express and purify the DAPDC protein, single colonies were picked from fresh transformation plates (LB agar containing ampicillin) and grown in 30 ml of LB medium containing ampicillin at 37°C while shaking at 200 rpm for 6 h. Then, 24 ml of the inoculum was used to inoculate 2.4 L of LB medium containing ampicillin and cultured to an OD of 0.4–0.5. The cells were induced with IPTG at a final concentration of 0.5 mM and continued to grow for another 12–14 h at 22°C while shaking at 100 rpm. After induction, the cultures were harvested by centrifugation at 6,000 × *g* for 15 min and washed three times with buffer A (20 mM Tris–HCl, 500 mM NaCl, 10 mM imidazole, 0.1 mM PLP, pH 8.0). Cells were finally suspended in 40 ml of buffer A and lysed by sonication on ice. Then, the cellular debris was centrifuged at 8,000 × *g* for 30 min, and the supernatant was collected and loaded on a pre-equilibrated 4-ml Ni-NTA column. The column was washed with 40 ml of buffer B (20 mM Tris–HCl, 500 mM NaCl, 40 mM imidazole, 0.1 mM PLP, pH 8.0). Finally, the recombinant protein was eluted with 10 ml of buffer C (20 mM Tris–HCl, 500 mM NaCl, 500 mM imidazole, 0.1 mM PLP, pH 8.0).

To avoid the effect of high concentrations of imidazole on enzyme activity, eluted fractions were further purified on a size exclusion CrystalDex G25 column equilibrated with gel filtration buffer (20 mM Tris–HCl, 100 mM NaCl, 0.1 mM PLP, 10% glycerol, pH 8.0). Fractions containing the protein of interest were identified by SDS-PAGE, and the BCA method was used to estimate the protein concentration.

### Expression and Purification of *Saccharomyces cerevisiae* Saccharopine Dehydrogenase (SDH)

To measure the enzymatic activity of *B. melitensis* DAPDC, the coupling enzyme SDH from *S. cerevisiae* is required. The pET30a plasmid containing the *S. cerevisiae* SDH protein coding gene was constructed by using the same process described above. The SDH expression and purification protocols were the same as those of pET21b-*lysA*, except the expression of SDH was conducted at 37°C. Fractions containing the SDH protein were identified by SDS-PAGE, and the BCA method was used to estimate the protein concentration.

### Optimization of the Conditions for the Coupled DAPDC-SDH Assay

The coupled DAPDC-SDH assay was optimized according to previously reported literature with proper modification (Peverelli and Perugini, 2015). In this assay, L-lysine, the product of DAPDC catalysis, was subsequently converted to saccharopine in a reaction coupled with α-ketoglutarate. The reaction was catalyzed by the coupling enzyme SDH and accompanied by the oxidation of NADH to NAD^+^, which was directly detected by monitoring the decrease in A_340 nm_.

All the enzymatic assays were conducted at room temperature. The enzymatic activity of SDH was optimized first. Prior to optimization of the SDH assay, a working stock of 10 mM NADH and a 500 mM lysine solution were prepared in deionized water, while a working stock of 500 mM α-ketoglutarate was prepared in 500 mM Tris buffer. Then, a mixture of NADH, α-ketoglutarate, L-lysine, and buffer (200 mM Tris, pH 8.0) was incubated with 50 ng of SDH in a final volume of 50 μl in 384-well plates. Reactions were mixed and incubated for 15 min. Twenty-five microliters of 8 M guanidine hydrochloride was added to terminate the reactions, and the A_340 nm_ was directly read in an EnVision multilabel plate reader. The decrease in A_340 nm_ was converted to the amount of NADH consumed using the equation of the regression line in an NADH standard curve. NADH standard curves were generated from serially diluted NADH at concentrations from 25 to 1,200 μM in 50 μl of SDH assay buffer (800 μM L-lysine, 4 mM α-ketoglutarate, 200 mM Tris–HCl, pH 8.0).

The DAPDC-SDH coupled assay was subsequently optimized. Prior to optimization of the DAPDC-SDH coupled assay, a working stock of 100 mM DAP was prepared, and hydrochloric acid was utilized to accelerate the dissolution of DAP. Enzyme reactions in the time course experiment were performed in a 50-μl volume of DAPDC assay buffer containing 200 mM Tris–HCl (pH 8.0), 4 mM α-ketoglutarate, 600 μM NADH, 0.1 mM PLP, and an excess amount of SDH (17 ng/μl). DAP at a saturating concentration (20 mM) and 50 ng of DAPDC were incubated in 384-well plates at room temperature. Reactions were terminated by the addition of 25 μl of 8 M guanidine hydrochloride over 3-min intervals, and A_340 nm_ was monitored. The optimal concentration of DAP for DAPDC activity was determined by performing the enzyme assay with DAP at various final concentrations ranging from 0 to 20 mM. The SDH and DAPDC activities are defined as micromoles (μM) of NADH consumed per minute per milligram of enzyme. GraphPad Prism 5.0 was used to analyze non-linear regression between the concentrations of NADH cofactor or substrates and the enzymes.

### HTS Assay of an FDA-Approved Drug Library to Identify *B. melitensis* DAPDC Inhibitors

The optimized DAPDC-SDH coupled assay described above was used to screen an FDA-approved drug library of 1,591 structurally diverse small-molecule drugs at a storage concentration of 10 mM dissolved in DMSO or water. The screening assay was carried out in transparent 384-well plates. Two hundred nanoliters of a compound was added to DAPDC reaction buffer at a final volume of 50 μl for a final concentration of 40 μM. Reactions were initiated by the addition of 50 ng of *B. melitensis* DAPDC. The enzyme-substrate-inhibitor mixture was then shaken at 1,000 rpm for 2 min and further incubated for another 16 min at room temperature. Then, 25 μl of 8 M guanidine hydrochloride was added to all plates to terminate the enzymatic reaction. The plates were shaken at 1,000 rpm for 2 min, and A_340 nm_ was then read using a PE EnVision microplate reader. Positive controls (enzyme in the reaction buffer) and negative controls (no enzyme in the reaction buffer) were also included in every reaction plate.

All the data from the screening assays were processed by using Microsoft Excel and GraphPad Prism 5.0 software. The inhibition rate was calculated by using the following equations:

(1) $$\%\mathrm{Inhibition}=\frac{\mu \,\left(\text{pos}\right)-\mathrm{Abs}\,\left(\mathrm{sam}\right)}{\mu \,\left(\text{pos}\right)-\mu \,\left(\text{neg}\right)}$$

(2) $$Z^{\prime }\,\mathrm{factor}=1-\frac{3\,\sigma \,\left(\text{pos}\right)+3\,\sigma \,\left(\text{neg}\right)}{\left|\mu \,\left(\text{pos}\right)-\mu \,\left(\text{neg}\right)\right|}$$

where Abs (sam) is the absorbance value of the compound tested, μ (pos) and σ (pos) are the mean absorbance value and standard deviation for the positive controls, respectively, and μ (neg) and σ (neg) are the mean absorbance value and standard deviation for the negative controls, respectively. The values of μ (pos), σ (pos), μ (neg), and σ (neg) used in the formula were based on data from 32 positive control wells or 32 negative control wells. In addition, the Z′ factor, a statistical coefficient value that utilizes the signal dynamic change (| μ (pos) − μ (neg)|) and data variability (3σ (pos) + 3σ (neg)) of reference controls to evaluate the quality of the HTS assay, was calculated. As shown in Eq. (2), the larger the difference between the negative and positive controls data and the smaller the signal variability, the closer the value of the Z′ factor is to 1, representing a higher quality of the assay.

Drugs with an inhibition rate greater than 40% were considered potential DAPDC inhibitors and used for further MIC determination.

### Determination of MIC Values Against *B. melitensis*

The MIC assay was carried out at the Chinese Center for Disease Control and Prevention that does not possess the *B. melitensis* NI strain. Because of the high degree of similarity (100%) between *B. melitensis* NI DAPDC and *B. melitensis* 16M DAPDC and the low interspecific difference in the genome of *Brucella* species, *B. melitensis* 16M stain was used in this MIC determination assay. The MIC assay against *B. melitensis* 16M was carried out in 96-well U-bottom plates by using standard procedures. In brief, the primary hits at a concentration of 10 mM were serially double diluted in 100-μl volumes of Mueller-Hinton broth. As a result, the drug concentrations ranged from 100 to 0.048 μM. Then, the turbidity of the bacterial inoculum was adjusted to match 0.5 McFarland standard, and the bacteria were inoculated with dilute drugs in a final volume of 200 μl. The plates were incubated at 37°C for 48 h. The lowest concentration of a drug that inhibited bacterial growth was determined and defined as the MIC.

## Results

### Phylogenetic and Comparative Analysis of *B. melitensis* DAPDC

The amino acid sequences of known DAPDC and eukaryotic ODC enzymes from different species were aligned and compared, and a phylogenetic tree was generated based on the alignment results. To develop potent drugs to treat brucellosis, the specific inhibitors against the DAPDC enzyme should not only possess antibacterial activity but also display low toxicity against mammals. Although the DAP/lysine biosynthetic pathway is absent in mammals, researchers have found that eukaryotic ODCs, which belong to the same group of PLP-dependent enzymes, show a certain degree of structural and mechanistic similarity with DAPDC (Fogle and Toney, 2011). Therefore, to explore the differences and evolutionary relationships between *Brucella* DAPDC and eukaryotic ODCs, *Homo sapiens* ODC and *S. cerevisiae* ODC were included in our analysis. The ODC enzyme in the polyamine biosynthetic pathway is responsible for catalyzing the decarboxylation of ornithine to putrescine. As shown in Supplementary Figure S1, *B. melitensis* DAPDC was more closely related to bacterial enzymes than to eukaryotic ODC enzymes (less than 30% sequence identity). Among DAPDC homologs, the DAPDC protein from *B. melitensis* shared the highest similarity with those from *Ochrobactrum* and *Bartonella* strains (93% sequence identity and 60% sequence identity, respectively) likely due to their similar survival conditions. In contrast, the *B. melitensis* DAPDC protein exhibited a lower identity to those of the enterobacterial strains *E. coli* and *S. enterica*, with sequence identities of 33 and 35%, respectively.

Multisequence alignment revealed conserved motifs among DAPDC and eukaryotic ODC enzymes (Supplementary Figure S2). In these enzymes, the active lysine residue in the KAFL motif is responsible for the covalent binding of these enzymes to PLP via formation of a Schiff base, whereas the EPGR and CESGD motifs are associated with subsequent substrate binding. The GGG motif is thought to interact with the phosphate group of PLP. Other conserved motifs, such as the HIGS motif, which is involved in protonation/deprotonation reactions, were also observed (Poulin et al., 1992; Gokulan et al., 2003).

### Expression and Purification of *B. melitensis* DAPDC

To biochemically characterize *B. melitensis* DAPDC, the pET21b-*lysA* and pET30a-SDH vectors were transformed into *E. coli* BL21 (DE3) cells. Prior to enzyme purification, the expression conditions were optimized to ensure high protein yields (data not shown). Then, the recombinant proteins were purified by Ni-chelating chromatography. An SDS-PAGE gel stained with Coomassie blue showed the steps of DAPDC purification (Supplementary Figure S3A). High expression levels of the His_6_-DAPDC protein were observed in the soluble fractions, and the recombinant DAPDC protein was purified and had an expected molecular weight (MW) of 40 kDa. In addition, the steps of *S. cerevisiae* SDH expression and purification were similar to those of DAPDC, except that the expression of SDH was carried out at 37°C. SDS-PAGE analysis also revealed the successful purification of soluble SDH protein with an expected MW of 41 kDa (Supplementary Figure S3B).

### Assay Optimization

**FIGURE 1:**
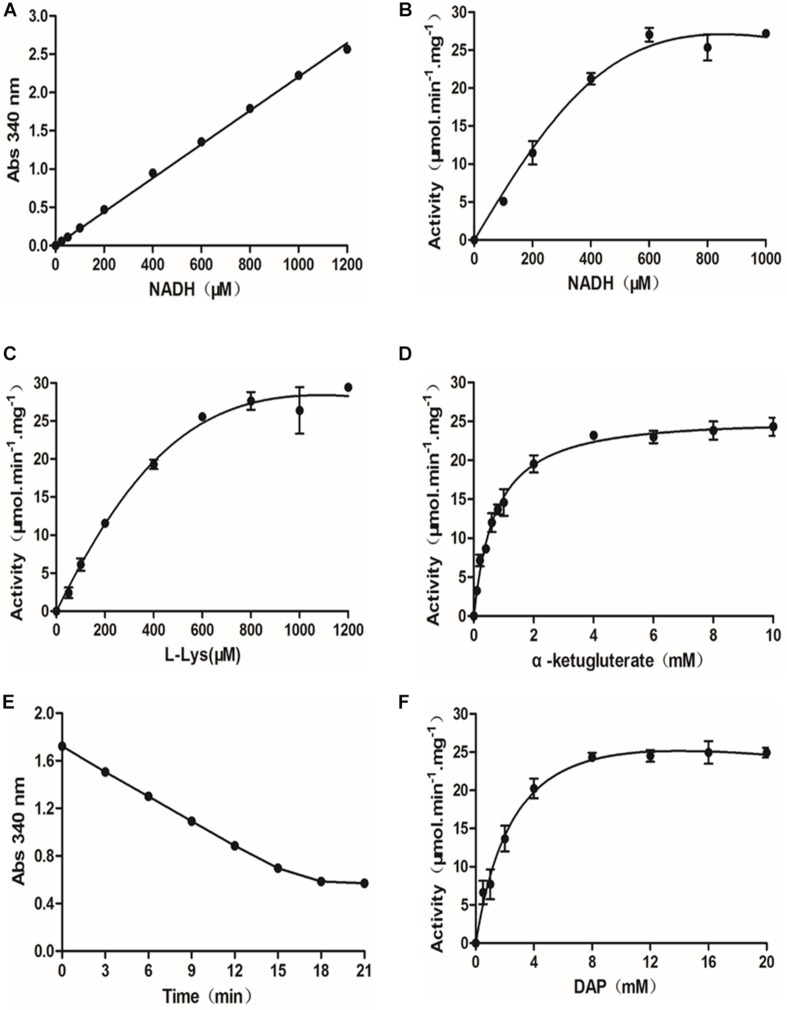
Optimization of the diaminopimelate decarboxylase (DAPDC)-saccharopine dehydrogenase (SDH) coupled assay. **(A)** Standard curve of NADH concentrations. NADH (25–1,200 μM) was serially diluted in SDH assay buffer, and the absorbance at 340 nm was directly read in a PE EnVision multilabel plate reader. The data are depicted as the means ± SDs obtained from three independent experiments. A robust linear correlation was observed between the absorbance of NADH and its concentration. **(B)** Dependence of SDH activity on the concentration of NADH. The activity of SDH was tested at various concentrations of NADH (0–1,000 μM). The values represent the means ± SDs of SDH activity determined from three independent experiments. **(C)** Activity of SDH as a function of the concentration of L-lysine. The activity of SDH at various concentrations of L-lysine (0–1,200 μM) was measured. The values depicted are the means ± SDs of SDH activity determined from three independent experiments. **(D)** Activity of SDH as a function of the concentration of α-ketoglutarate. The activity of SDH at various concentrations of α-ketoglutarate ranging from 0 to 1,200 μM was measured. The values depicted are the means ± SDs of SDH activity determined from three independent experiments. **(E)** Time curves for the DAPDC reaction. The DAPDC activity was measured based on conditions optimized for SDH. Fifty nanograms of DAPDC was incubated with 20 mM DAP in DAPDC assay buffer containing 200 mM Tris–HCl (pH 8.0), 4 mM α-ketoglutarate, 600 μM NADH, 0.1 mM PLP, and an excess amount of SDH (17 ng/μl) in a total volume of 50 μl. Reactions were terminated by the addition of 25 μl of 8 M guanidine hydrochloride over a 3-min interval, and the absorbance at 340 nm was monitored. The data are depicted as the means ± SDs of Abs_340 nm_ values obtained from three independent experiments. **(F)** Activity of DAPDC as a function of the concentration of DAP. The activity of DAPDC was tested at various concentrations of DAP (0–20 mM). The values depicted are the means ± SDs of DAPDC activity determined from three independent experiments.

To develop a quantitative SDH-DAPDC coupled assay and adapt it for use in a 348-well format, a standard curve was established from the absorbance of NADH at multiple concentrations in a total volume of 50 μl at 340 nm. As shown in Figure 1A, the standard curve exhibited excellent linearity in the range of 0–1,200 μM NADH. Moreover, the addition of 25 μl of 8 M guanidine hydrochloride had no effect on the absorbance of NADH at 340 nm (data not shown). Thus, based on the observed linear relationship, we set out to optimize the assay conditions for measuring the activity of SDH. In time course curves prepared with 50 ng of SDH, the fairly linear steady-state decrease in NADH persisted for up to 15 min after the start of the reaction (Supplementary Figure S4). In addition, the decrease in A_340 nm_ was close to 1.0, providing a clear signal with which to monitor the reaction. Therefore, a reaction time of 15 min was chosen to ensure that further measurements were conducted under conditions in which the reaction velocity was first order and a robust signal was thus observed. Then, the influence of various parameters, such as the concentrations of the NADH cofactor and substrates (L-lysine and α-ketoglutarate), on SDH activity was evaluated. When measured as a function of the concentration of NADH (0–1,000 μM), SDH exhibited maximal activity with NADH at a concentration near 600 μM (Figure 1B). Thus, a concentration of 600 μM was determined to be the optimal NADH concentration in the coupled assay. The concentrations of the substrates in the SDH reaction, lysine and α-ketoglutarate, were also optimized by varying the substrate concentration and monitoring the SDH activity in the presence of 600 μM NADH and 50 ng of SDH. As shown in Figures 1C,D, the activity of SDH reached the plateau phase when the concentrations of the substrates L-lysine and α-ketoglutarate were 800 μM and 4 mM, respectively.

The optimized SDH assay provides a method to quantitate the conversion of lysine, the product of DAPDC, into saccharopine. Thus, we set out to determine DAPDC activity on the basis of conditions optimized for SDH. First, a time course curve containing 50 ng of DAPDC and DAP at 20 mM, a saturating concentration, was conducted to determine the reaction time. In the coupled enzyme assay, excess SDH was added to DAPDC reactions. As shown in Figure 1E, the amount of NADH consumed was fairly linear until 18 min from the start of the reaction. We also observed the hyperbolic behavior of DAPDC activity as a function of DAP concentration ranging from 0.5 to 20 mM (Figure 1F). DAPDC activity peaked when DAP was at a concentration near 8 mM. Therefore, the optimal conditions for further HTS assays were a reaction mixture containing 200 mM Tris, pH 8.0, 0.1 mM PLP, 600 μM NADH, 4 mM α-ketoglutarate, and 10 mM DAP and a reaction time of 18 min.

### HTS Assay Inhibitors of the *B. melitensis* DAPDC Enzyme

**FIGURE 2:**
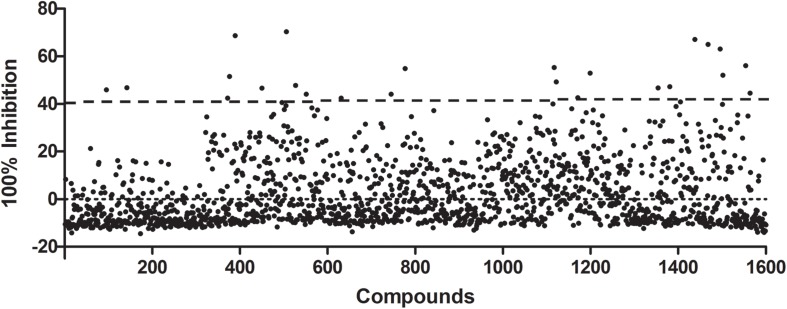
Preliminary studies of diaminopimelate decarboxylase (DAPDC) enzyme activity inhibition using a US Food and Drug Administration (FDA)-approved drug library. Each compound was tested at a final concentration of 40 μM. Twenty-four compounds exhibited an inhibition rate greater than the cutoff value of 40%, representing a hit rate of 1.5%. All data were processed by using Microsoft Excel and GraphPad Prism 5.0 software.

**FIGURE 3:**
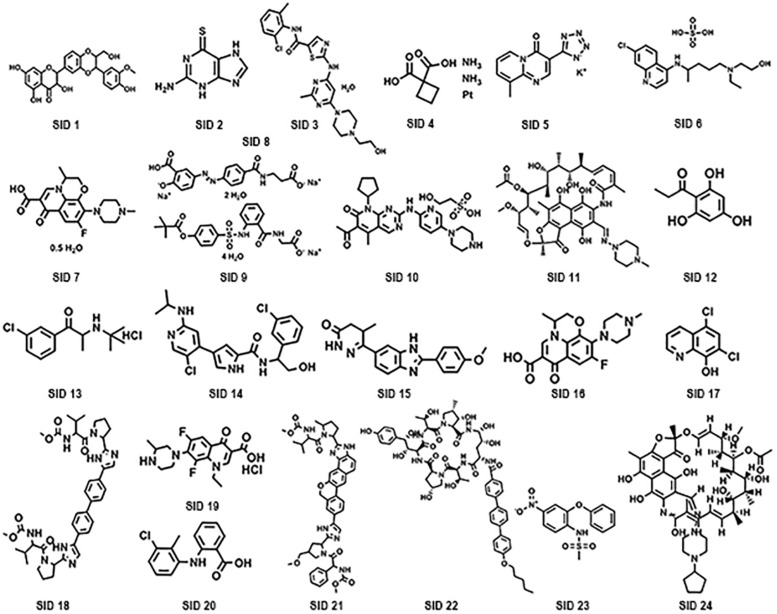
Chemical structures of potential diaminopimelate decarboxylase (DAPDC) inhibitors identified from the primary screen.

A subset of 1,591 low-molecular-weight compounds accessible from an FDA-approved drug library was selected for HTS assays. Using the optimized conditions described above, each reaction was conducted in 384-well transparent plates in a total volume of 50 μl. The cutoff for the HTS assay was set to 40%, and 24 compounds at a concentration of 40 μM exhibited inhibitory activity against the DAPDC enzyme, representing a hit rate of 1.5% (Figure 2). Among the compounds from our primary screening assay, SID1, SID2, SID3, SID4, and SID5 were the most potent drugs, with inhibition rates of 60–70%. The enzymatic activity of DAPDC was also inhibited by 40–60% in the presence of 19 other compounds, with SID24 exhibiting the least potent inhibitory effect on DAPDC enzymatic activity (Table 1). The diverse structures of the primary hits are shown in Figure 3. The Z′ factor, a statistical parameter used to evaluate the suitability of the HTS assay, was also calculated (Zhang et al., 1999). The HTS assay carried out as described above produced a Z′ value of 0.72, which was greater than 0.5, the benchmark used to evaluate HTS assays as excellent.

**TABLE 1:** The chemical characteristics, inhibitory rates, and minimum inhibitory concentration (MIC) values of primary hits.

**Compound ID**	**Selleck number**	**MW**	**Inhibitory rate (%)**	**MIC (μM)**
SID1	S2357	482.44	70.30	NS^a^
SID2	S1774	167.19	68.70	NS^a^
SID3	S7782	506.02	67.09	100 μM
SID4	S1215	371.25	65.03	0.20 μM
SID5	S4008	266.3	63.08	NS^a^
SID6	S4430	433.95	56.09	NS^a^
SID7	S4604	370.38	55.29	Excluded^b^
SID8	S3745	437.31	54.83	NS^a^
SID9	S4666	528.51	52.88	NS^a^
SID10	S1579	573.66	52.08	NS^a^
SID11	S1764	822.94	51.50	Excluded^b^
SID12	S4249	182.17	49.21	NS^a^
SID13	S2452	276.2	47.72	NS^a^
SID14	S7854	433.33	47.26	25 μM
SID15	S1550	334.37	46.80	50 μM
SID16	S1940	361.37	46.69	Excluded^b^
SID17	S1839	214.05	46.58	Excluded^b^
SID18	S1482	738.88	45.89	Excluded^b^
SID19	S2539	387.81	44.51	Excluded^b^
SID20	S1959	261.7	44.05	25 μM
SID21	S3724	883	44.05	NS^a^
SID22	S4286	1, 140.24	42.56	NS^a^
SID23	S2040	308.31	42.45	NS^a^
SID24	S1760	877.03	42.45	Excluded^b^

*^*a*^NS, not sensitive; ^*b*^Excluded: compounds not tested by MIC determination assay.*

### MIC Determination

According to the results of the HTS assays, the enzymatic activity of DAPDC was inhibited by 24 compounds. However, among these primary hits, seven compounds [such as SCID7 (levofloxacin) and SCID11 (rifampin)] were antibiotics that have been widely used in the clinical treatment of bacterial infections. In view of this, the remaining 17 compounds were selected for further evaluation of their potential bacteriostatic effect against *Brucella*. The compounds were used in the MIC assay at concentrations ranging from 0.048 to 100 μM. As shown in Table 1, *Brucella* growth was inhibited by five compounds (SID3, SID4, SID14, SID15, and SID20), the MIC of which ranged between 0.2 and 100 μM. Compound SID4 exhibited the most potent activity against *Brucella* with an MIC value of 0.2 μM, and compounds SID3, SID14, SID15, and SID20 possessed less antibacterial activity than compound SID4. The MICs of the other primary hits could not be detected even at the highest concentration tested of 100 μM.

## Discussion

In a previous study carried out by our laboratory, 10,832 mutants in a transposon mutant library of *B. melitensis* were successfully defined as insertion sites for transposons inserted throughout the genome. However, 948 genes exhibited no detectable transposon insertions, suggesting that some of these genes are essential *in vitro* or *in vivo* (De et al., 2017). One of these genes with no detectable transposon insertions was the *lysA* gene of *B. melitensis*. To detect the essentiality of the *lysA gene*, construction of a *lysA* deletion mutant of *B. melitensis* was attempted using the homologous recombination method. However, the deficient phenotype was not obtained in at least three attempts, indirectly implying that the *lysA* gene might play an essential role in the survival of *B. melitensis*.

The L-lysine biosynthetic pathway, which is absent in mammals, is an attractive target for the development of novel antibacterial agents. Several inhibitors to target the enzymes that participate in this pathway have been developed. Based on analogy to pyruvate, α-ketopimelic acid was indicated to inhibit dihydrodipicolinate synthase (DHDPS), which is essential for mycobacterial growth (Shrivastava et al., 2016). For the treatment of brucellosis, the screened antibacterial compounds should not only possess the ability to kill pathogens but also have little or no effect on the intestinal flora and the health of the host. Analysis of the amino acid sequences of DAPDC homologs from different species highlights the distant relationship and low degree of identity between DAPDC from *Brucella* and that from the enterobacterial strains *E. coli* and *S. enterica* and human ODC (33, 35, and 28% sequence identity, respectively). In addition, compared with the GGG, HIGS, and EPGR motifs, the KAFL and CESGD motifs were less conserved among these species. The results of bioinformatics analysis suggested that the screened inhibitors of *Brucella* DAPDC may have little effect on other species.

In this study, recombinant DAPDC from *Brucella* was expressed as soluble protein, which might have largely maintained its natural function and structure. Lysine and carbon dioxide are produced as products of DAPDC catalysis; however, neither of these compounds are directly applicable to high-throughput detection. In this assay, a DAPDC-SDH coupled method was employed to determine the activity of DAPDC. The production of lysine by DAPDC was coupled to the detection of NADH oxidation by the catalysis of SDH. The reaction was easily monitored spectrophotometrically by detecting the decrease in A_340 nm_. To better adopt this coupled assay for HTS assays, the coupled assay was optimized in a final volume of 50 μl. Various parameters (NADH, α-ketoglutarate, and DAP concentrations and reaction time) were optimized to ensure that the reactions were sensitive to further inhibition. Moreover, to conveniently detect the reaction, 8 M guanidine hydrochloride was used to terminate the enzyme reaction instead of monitoring the A_340 nm_ as a function of time.

After the HTS assay with an FDA-approved drug library containing 1,591 compounds was carried out, 24 primary hit compounds were identified based on their degree of reaction. Among these compounds, five drugs (SID3, SID4, SID14, SID15, and SID20) could inhibit the growth of *B. melitensis in vitro*. Moreover, we also evaluated the cross-reactivity of these inhibitors with the enterobacterial species like *E. coli* (ATCC 25922) and *S. enterica* (GIM 1.712) by MIC assay. None of the five compounds were active against the *E. coli* and *S. enterica*, even at the concentration of 100 mM (data not shown). The result suggested that the compounds we screened are highly specific in their ability to kill *Brucella* and further validated our bioinformatics analysis of the *Brucella* DAPDC. On the other hand, all of the five compounds are proven to be safe and successfully applied in the treatment of human diseases. Among them are SID 3 (dasatinib monohydrate), a new kind of multitargeted inhibitor of several critical oncogenic kinases for the treatment of thyroid cancer (Roy et al., 2012); SID4 (carboplatin), a kind of antineoplastic agent used mainly for the treatment of human cancer via interfering with DNA synthesis (Banerji et al., 2008); SID14 (ulixertinib, BVD-523), a highly potent, selective, and reversible ERK1/2 inhibitor used for the treatment of advanced solid tumors (Mendzelevski et al., 2018); SID 15 (pimobendan), a phosphodiesterase III inhibitor used for the treatment of chronic and acute heart failure (Iwasaki et al., 1999); and SID 20 (tolfenamic acid), a traditional anti-inflammatory compound that acts as a kind of cyclooxygenase inhibitor commonly used for the treatment of inflammation-related diseases (Feldman et al., 2018). However, researchers recently found new applications for cyclooxygenase inhibitors, such as celecoxib, that possess bactericidal effects against some pathogenic bacteria (such as *Staphylococcus aureus*, *Bacillus anthracis*, and *Pseudomonas aeruginosa*) (Chiu et al., 2012; Thangamani et al., 2015). The new application of conventionally approved drugs has recently received increasing attention. Auranofin, which is applied for the treatment of human rheumatoid arthritis, was found to possess inhibitory activity against parasites (such as *Trypanosoma brucei* and *Leishmania)* and bacteria (Harbut et al., 2015; Da et al., 2016). Compared with the development of novel drugs, this strategy has the advantages of a lower cost, increased speed, and the availability of clear and detailed drug information. The five compounds used to treat human diseases identified in our study were also found to exhibit bactericidal activity against *Brucella*, a successful example of a new use for old drugs. Furthermore, our data suggested that the *Brucella* DAPDC serves as a useful target for innovative antibacterial therapy and that the five identified compounds can be used to treat brucellosis with proper modification.

## Data Availability Statement

Publicly available datasets were analyzed in this study. This data can be found here: WP_011004944.1, NP_417315.1, WP_003406632.1, WP_077231782.1, WP_000216946.1, WP_000216946.1, WP_000216946.1, WP_078690242.1, WP_006467923.1, WP_000056576.1, WP_004398851.1, NP_012737.1, and NP_001274118.1.

## Author Contributions

QW and PB conceived the idea, designed the experiment, and wrote the manuscript. PB, XY, and CZ performed the research and generated the data.

## Conflict of Interest

The authors declare that the research was conducted in the absence of any commercial or financial relationships that could be construed as a potential conflict of interest.

## Abbreviations

A_340 nm_: absorbance at 340 nm
DAP: diaminopimelate
DAPDC: diaminopimelate decarboxylase
HST: high-throughput screening
IPTG: isopropyl β-D -galactopyranoside
OD: optical density
ODC: ornithine decarboxylase
PLP: pyridoxal 5′-phosphate
SDH: saccharopine dehydrogenase.
