# Supplementary material for: Identification of Small-Molecule Inhibitors of Brucella Diaminopimelate Decarboxylase by Using a High-Throughput Screening Assay
Source: Front Microbiol. 2020 Jan 21;10:2936. doi: 10.3389/fmicb.2019.02936 (PMC6986272; doi:10.3389/fmicb.2019.02936)
Supplement: Supplementary file 1 [file Data_Sheet_1.docx]

Supplementary Material

Identification of small molecule inhibitors of *Brucella* diaminopimelate decarboxylase by a high-throughput screen assay

Pengfei Bie^1^, Xiaowen Yang^1, 2#^, Cunrui Zhang^1^^, 3#^ and Qingmin Wu^1^*

1. Department of Preventive Veterinary Medicine, College of Veterinary Medicine, China Agricultural University, Beijing, China

*** Corr**e**spondence:**

Qingmin Wu

wuqm@cau.edu.cn

**^#^ Present address:**

2. State Key Laboratory for Disease Prevention and Control, Collaborative Innovation Center for Diagnosis and Treatment of Infectious Disease, National Institute for Communicable Disease Control and Prevention, Chinese Center for Disease Control and Prevention, Beijing, China

3. China Animal Disease Control Center, Beijing, China

Table of contents:

Supplementary Figure S1. Phylogenetic analysis of DAPDC homologs and eukaryotic ODC enzymes.

Supplementary Figure S2. Multiple sequence alignment analysis of the DAPDC family and eukaryotic ODC enzymes.

Supplementary Figure S3. Purification of *B. melitensis* DAPDC and *S. cerevisiae* SDH.

Supplementary Figure S4. Time curves for the SDH reaction.

Table S1. Primers used in this work.

| Primer name | Sequence (restriction enzyme)(5′-3′) | Locus (gene) |
| --- | --- | --- |
| Primers used for construction of the DAPDC mutants. | | |
| Upstream-F | GCGAGCTCGATCTGGCAAAGCTCTCG (*Sac*I) | BMNI I1887 upstream |
| Upstream-R | AGTGGGTAGAATTCGGGAAATGTCCCTTGAGG (*EcoR*I) | BMNI I1887 upstream |
| Downstream-F | GAATTCATCCCACTATTATAATTCTCATGGC (*EcoR*I) | BMNI I1887 downstream |
| Downstream-R | GCGGATCCGATCATGCGTGCCGGTGG (*BamH*I) | BMNI I1887 downstream |
| Kan-F | GCGAATTCCACGTCTTGAGCGATTGTGTA (*EcoR*I) | Kan cassette |
| Kan-R | GCGAATTCGATTCCGAAGCCCAACCTT (*EcoR*I) | Kan cassette |
|  |  |  |
| Primers used for construction of the pET21b-*lysA* expression vector | | |
| DAPDC-F | GCGGATCCGGTGAACCACTTTGAATATCGC (*BamH*I) | BMNI I1887 |
| DAPDC-R | GCCTCGAGTTACAGCCAGTCCGGCAC (*Xho*I) | BMNI I1887 |
|  |  |  |
| Primers used for construction of the pET30a-SDH expression vector | | |
| SDH-F | GCGGATCCATGGCTGCCGTCACATTACAT (*BamH*I) | YIR034C |
| SDH-R | GCCTCGAGCTACAATCTTGAAGATCTTTTAACA (*Xho*I) | YIR034C |

**Table S1.** Primers used in this work.

The primers used for the construction of the DAPDC mutants, the pET21b-*lysA* expression vector and the pET30a-SDH expression vector were listed in the Table S1.

**

**

**Figure S1.** Phylogenetic analysis of DAPDC homologs and eukaryotic ODC enzymes. The phylogenetic tree was generated using MEGA 5 software with the neighbor-joining method, and evolutionary distances, which are shown as the number of amino acid substitutions per site, were computed with the Poisson correction method. The analysis involved 13 amino acid sequences. Branches are labeled with the protein accession number along with organism name and similarity. The identity was calculated by comparing the amino acid sequence of *B. melitensis* DAPDC with that of other DAPDC homologs and eukaryotic ODC enzymes.


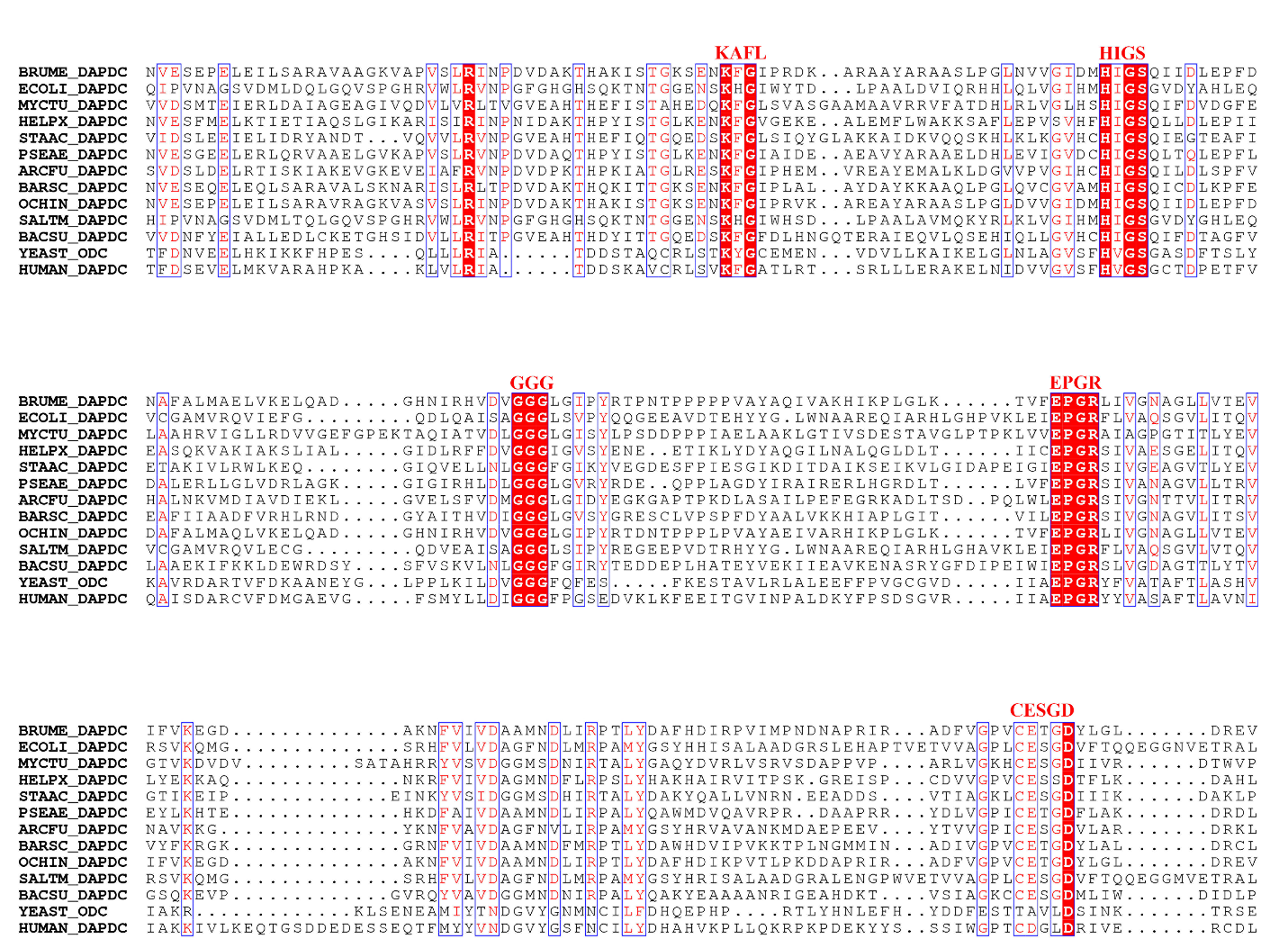


**Figure S2.** Multiple sequence alignment analysis of DAPDC family members and eukaryotic ODC enzymes. The alignment was carried out using ClustalW software. The amino acid residues which are considered as highly similar are colored in red and framed in blue. Moreover, the conserved motifs are also indicated above the relevant residues.

**
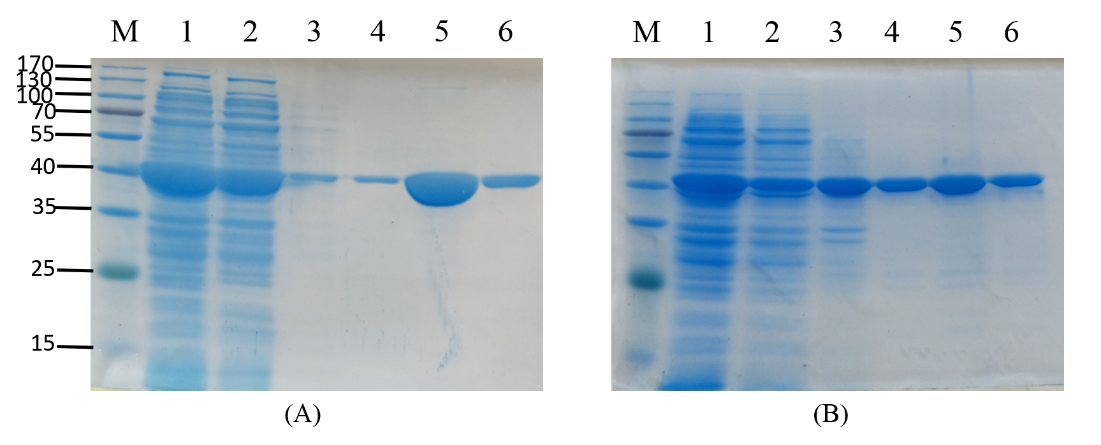
**

**Figure S3.** Purification of *B. melitensis* DAPDC and *S. cerevisiae* SDH. The His-tag recombinant proteins were purified as described in the Materials and Methods section and analyzed on 10% SDS-PAGE gels stained with Coomassie blue. (A) Steps of DAPDC purification. Lane M, molecular mass marker. Lane 1, sonicated lysates of induced *E. coli* BL21 (DE3) cells expressing *B. melitensis* DAPDC. Lane 2, the flow-through from Ni-NTA column chromatography. Lane 3, the eluates during purification (elution with 40 mM imidazole). Lanes 4 to 6, purified fractions of eluted DAPDC (elution with 500 mM imidazole). Lane 5, the peak fraction of purified DAPDC with an expected MW of 40 kDa. (B) Steps of SDH purification. Lane 1, sonicated lysates of induced *E. coli* BL21 (DE3) cells expressing *S. cerevisiae* SDH. Lane 2, the flow-through from Ni-NTA column chromatography. Lane 3, the eluates during purification (elution with 40 mM imidazole). Lanes 4 to 6, purified fractions of eluted SDH (elution with 500 mM imidazole). Lane 5, the peak fraction of purified SDH with an expected MW of 41 kDa.


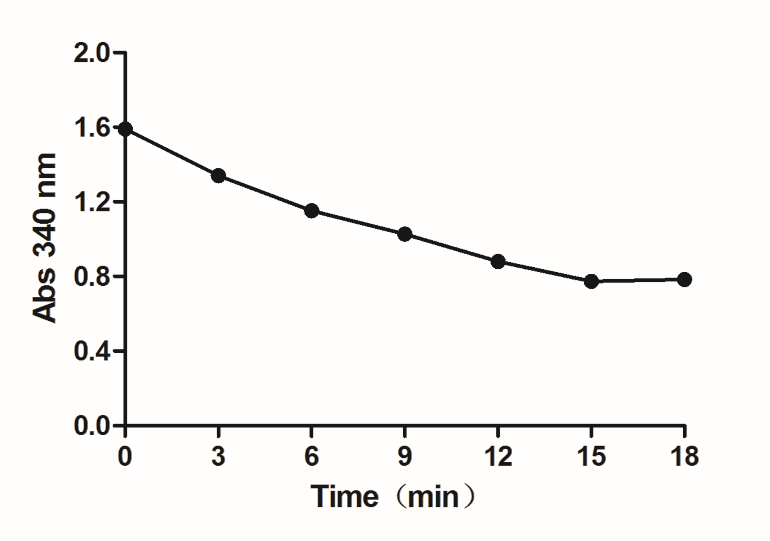
**Figure S4.** Time curves for the SDH reaction. Enzyme reactions were carried out in a 50 μl volume containing 800 μM L-lysine, 4 mM α-ketoglutarate, 200 mM Tris-HCl (pH 8.0), and 600 μM NADH. Reactions were initiated by the addition of 50 ng of SDH and quenched by the addition of 25 μl of 8 M guanidine hydrochloride at 3 min intervals. Then, the absorbance at 340 nm was directly measured. The data are depicted as the means ± SDs of Abs _340 nm_ values obtained from three independent experiments.
